# High Actin Expression in Thrombus of Acute Ischemic Stroke Can Be a Biomarker of Atherothrombotic Origin Stroke

**DOI:** 10.3389/fneur.2022.896428

**Published:** 2022-07-22

**Authors:** Rongyu Wang, Zhiqiang Wang, Lianyan Jiang, Gangfeng Gu, Bo Zheng, Liulin Xian, Yaodan Zhang, Jian Wang

**Affiliations:** ^1^Department of Neurology, Hospital of Chengdu University of Traditional Chinese Medicine, Chengdu, China; ^2^Department of Neurology, Chengdu BOE Hospital, Chengdu, China; ^3^Department of Neurology, Ya'an People's Hospital, Ya'an, China; ^4^Acupuncture and Tuina College, Chengdu University of Traditional Chinese Medicine, Chengdu, China

**Keywords:** acute ischemic stroke, thrombus, stroke etiology, actin, CD105

## Abstract

**Background:**

As the treatment target, the imaging information and histologic characteristics of the thrombus may differ according to the stroke subtype. This study aimed to provide the correlative study of stroke etiology with the non-contrast CT, and histological composition of retrieved clots in acute ischemic stroke (AIS).

**Materials and Methods:**

A total of 94 patients with AIS who underwent the endovascular treatment with successfully retrieved clots from January 2017 to October 2020 were enrolled in the present study. Histological analysis was performed using hematoxylin and eosin (H&E) staining and immunostaining with CD3, CD20, CD105, and actin antibodies. CT obtained at the patients' admission was to measure the attenuation and volume of all thrombus.

**Results:**

A total of 94 subjects were included in this study. Fifty-six patients were classified as cardioembolic (CE), and 38 were classified with large-artery atherosclerosis (LAA). The subjects with LAA tend to exhibit higher actin and CD105 levels, and lower Hounsfield Unit (HU) values than subjects with CE. After adjusting for confounders, the actin was positively correlated with CD105 but not with HU values. Logistics regression shows actin was valuable for the prediction of LAA (OR, 1.148; 95% CI, 1.075–1.227; *p* < 0.001), even adjusted for age, sex, and intervention type (OR, 1.129; 95% CI, 1.048–1.216; *p* = 0.001), CT density and CD105 (OR, 1.161; 95% CI, 1.056–1.277; *p* = 0.002). Actin levels have a strong accuracy in differentiating LAA from CE, especially combined with CT density and CD105, which yielded a sensitivity of 63.2%, a specificity of 89.3%, with the area under the curve (AUC) at 0.821 (95% CI, 0.731–0.912).

**Conclusion:**

Our findings suggest that actin's level was a major factor differentiating atherothrombotic origin strokes from the cardioembolic stroke.

**Clinical Trial Registration:**

**ChiCTR2100051173**.

## Introduction

Stroke is the leading cause of disability and mortality, influencing more than 100 million humans worldwide (1–3). As one of the most common types of stroke, acute ischemic stroke (AIS) accounts for 60–80% of strokes, which is usually caused by the obstruction of large vessels. Reperfusion, such as intra-arterial thrombectomy (IAT) and intravenous thrombolysis (IVT), is the most effective therapy that allows the timely recanalization of occluded vessels. Given the fact that the progress in mechanical thrombectomy (MT) makes the histologic analysis of retrieved thrombus available, there has been an increasing interest in the analysis of the association between clots composition and stroke subtype. Non-contrast computed tomography (NCCT) is the first choice for patients with suspected stroke in clinical. Understanding thrombus composition and its relation with stroke subtypes and CT information could help gain more insight into the etiopathogenesis of stroke and potentially influence therapy decisions (4–7). The composition of the thrombus may affect the thrombolytic efficacy of recombinant tissue plasminogen activator (rtPA) because red (erythrocyte-rich) clots are considered to have a better clinical outcome than white (platelet-rich) clots (8). Even if clot composition is unavailable before MT, pre-interventional imaging information such as CT and MRI may help us know about the components of thrombus. Therefore, their association with each other is essential for patients with AIS. If there is no such link, it would suggest that more advanced analyses such as microRNA signatures and proteomic analysis should be considered to elucidate the composition of the thrombus (9).

Thrombus characteristics are complicated and various, including the thrombotic factors such as fibrin and platelets, the inflammation process of leukocytes infiltration, and the participation of endothelial cells (10). The formation of the thrombus is closely interlinked with the inflammation process. Once there is an endothelial injury, platelets will be bound to damaged vessels, thus causing the formation of a thrombus (11). Thrombus formation is characterized by the presence of inflammatory cell infiltrate mainly composed of T lymphocytes and B lymphocytes that modulate the thrombus formation process by secreting inflammatory mediators (12). Furthermore, platelets would induce B-cell switching and T-cell responses, and aggregate the lymphocytes to the sites of injured vessels (13, 14). However, thrombus composition in different stroke subtypes is unclear, and its relation to imaging information has not reached a consensus (15–18).

Previous studies suggest that stroke may also be a manifestation or complication of hematological disorders (19). A series of changes in blood composition may occur before a stroke. For example, in polycythemia vera, approximately half of all patients also present thrombocytosis and leukocytosis. High viscosity resulting from increased hematocrit plays a significantly important role in the pathogenesis of thrombosis (20). Thus, there exists a strong correlation between hematocrit and thrombosis rate. Increased activation of leukocytes and platelets and their interaction with endothelial cells are also considered to be potential contributing factors to thrombosis. In addition to platelet activation and microparticle release, leukocyte activation and neutrophil activation in essential thrombocythemia may also play a role in thrombosis. Therefore, analyzing the components of thrombus may also help us understand the concomitant conditions of other diseases and could serve as a reason to intensify diagnostic workup for hematological disorders in these patients.

In this study, immunohistochemical staining was used to label T lymphocytes (CD3) and B lymphocytes (CD20), as well as endothelial cells (CD105) and actin, to clarify the role of these cells in thrombosis and the identification of stroke types. Although many studies have been published to analyze the relationship between imaging information, histologic characteristics of thrombus, and stroke subtypes, they mainly focus on traditional hematoxylin and eosin (H&E) staining and Martius scarlet blue (MSB) staining to examine the clots' composition. Only a few studies evaluated the role of immunohistochemistry (IHC) analysis. Thus, we perform the correlative study of stroke etiology with the CT density and histopathologic analysis of retrieved thrombus in AIS, further providing more insight into stroke's etiopathogenesis.

## Materials and Methods

A prospective-multicenter cross-sectional study was conducted to evaluate the relationship between the CT density and thrombus IHC information with stroke etiology of patients with AIS secondary intracranial vessel occlusion who underwent MT from January 2017 to October 2020. Patients who meet all the following criteria could be involved: age >18, cerebrovascular occlusion, a CT scan before MT procedure, successful MT, and signed informed consent. Patients would be excluded if they missed key data about the study or if their thrombotic materials were unsuitable for histopathologic analysis. In this study, all the following data will be collected, including demographic features (age and sex), cerebrovascular risk factors (hypertension, diabetes, hyperlipidemia, atrial fibrillation, and smoking), occlusion location [middle cerebral artery (MCA), internal carotid artery (ICA), common carotid artery (CCA), basilar artery (BA), vertebral artery (VA)], intervention types, and the stroke subtypes were classified following the Trial of ORG10172 in Acute Stroke Treatment (TOAST) criteria (21), which was illustrated as follows: large artery atherosclerosis (LAA), cardioembolism (CE), small-vessel occlusion, other determined etiology, or undetermined etiology.

### Non-Contrast CT Analysis

Non-contrast CT (NCCT) was used to detect the thrombus density (22), which was measured in Hounsfield Unit (HU). The density and volume were measured as previous studies described (23, 24), which can be briefly described as follows: two board-certified vascular neurologists with accreditation in neuroimaging blinded to clinical characteristics and histopathologic features reviewed the NCCT sequences. The HU value is calculated by the sum of each section's region of interest (ROI) and by dividing the number of sections, and excluded regions that represent calcifications with more than 100 HU values. Philips Brilliance CT 64-channel scanner with parameters of 120 kVp, 300 mAs, and a 1-mm reconstructed slice thickness was set. Thrombus volume was calculated using π, the radius of the major axis and the minor axis, and the height of the cylinder according to the formula of the cylinder with an elliptical base.

### Mechanical Thrombectomy Procedure

The MT procedure was carried out in a biplane neuro-angiography suite (Axiom Artis, Siemens, Munich, Germany) by experienced neuro-interventional radiologists with more than 5 years of experience (25). All patients were under general anesthesia with intravenous fentanyl. Aspiration with the A Direct Aspiration First-Pass Thrombectomy (ADAPT) technique, stent retriever with Solumbra technique, or a combination could be accepted. The ADAPT technique was performed using a large-bore aspiration catheter such as the 5 Max ACE or ACE 64 (Penumbra system), with 0.016 microwires in a microcatheter as an introducer to the clot face. The Solumbra technique consisted of deploying a stent retriever (Solitaire, Trevo, or Mindframe) distal to the clot.

### Histopathologic Features Analysis

The retrieved thrombus was immediately fixed in phosphate-buffered formalin and embedded in paraffin. Then, it was cross-sectioned at the 4-μm thickness and stained with H&E staining. The IHC was performed to detect and quantify CD3 for T cells, CD20 for B cells, CD 105 for endothelial cells, and actin expression (26). Leica DM500 microscope and digital camera (Leica, Germany) were used to randomly take photos (200 magnification) of stained slices in at least three fields. Those photos then were transformed into digital images [Tag Image File Format (TIFF) or Joint Photographic Experts Group (JPEG)] for further histological quantification by Image-Pro Plus 6.0 software following the standard operating procedure (27). The slices with the largest area of brown-yellow color were selected, and each image was analyzed to obtain the cumulative integrated optical density (IOD) of each photo and the pixel area (AREA) of the tissue and then calculate the areal density (IOD/AREA), the value of which represents the expression level of each composition. All those procedures were performed by a pathologist blinded to the patient's clinical information.

### Statistical Analysis

The continuous variables are represented by the mean ± standard deviation (SD) or the median [interquartile range (IQR)] and are compared using Student's *t*-test or the Mann–Whitney U test in each group. The discrete variables data are represented by the percentage (%) and are compared using the chi-square test or Fisher's exact probability method. IHC staining cells and histopathology results are compared using an ANOVA test, and Dunnett's T3 test was used for *post-hoc* comparison. The Spearman rank correlation was used to test the significant correlations between imaging information and histopathologic analysis. Partial correlation was used to further examine the correlation between them after adjusting for age, sex, intervention type, and other clots composition. Multiple logistic regression analysis was performed to identify the independent factors of discriminating LAA from the CE subtype. The receiver operator characteristic (ROC) curve was drawn to assess the accuracy of independent factors to differentiate the LAA. Maximizing the Youden index was adopted to define the threshold of variables. When *p* < 0.05, the difference was statistically significant. All statistical analysis was conducted using IBM^®^SPSS^®^Statistics software (Version 21; USA).

## Results

A total of 107 consecutive patients at five centers who underwent MT with intracranial artery occlusion were considered eligible during the study period. After the exclusion of patients who missed key data (*n* = 6) and those whose thrombotic materials are unsuitable for histopathologic analysis (*n* = 7), a total of 94 patients with AIS (mean age: 64 ± 21 years; 46 male) qualified for the following analysis. The most frequent vascular risk factor is hypertension (58/94). Angiography demonstrated that the MCA occlusion shows the highest frequency among 40 patients. The median NIH stroke scale (NIHSS) score on admission was 17, with scores ranging from 8 to 26. Across all retrieved thrombus, 10 were retrieved by ADAPT technique, 20 by stent retrieved, and 64 by a combination of aspiration and stent retrieval. For NCCT analysis, the median thrombus attenuation was 50 (9), and the volume was 0.10 (0.21). For histopathological analysis, 12 of the retrieved thrombi were classified as red clots [red blood cell (RBC) dominant], 62 were classified as mixed clots (RBC equal to fibrin), 10 were classified as white clots (fibrin dominant), and the other 10 were classified as organized clots.

The baseline characteristics, imaging information, and histologic features of the 94 patients in the LAA and CE subtypes were shown in Table 1. Among the 94 subjects, 56 patients were classified as cardioembolic origin and 38 of atherothrombotic origin. No other stroke etiology was found in this study. As shown in Table 1, the thrombus HU value was significantly lower in the LAA group when compared to the CE group (48 *vs*. 52, *p* = 0.010). IHC staining results show that patients with the LAA source had significantly higher actin and CD105 levels than patients with the CE source (17.46 *vs*. 8.16, *p* < 0.001, 16.54 *vs*. 9.46, *p* = 0.001, Table 1). Both CD105 and actin levels were found to be higher than CD3 and CD20 levels in each group. Thrombus of LAA and CE source was most likely to be RBC equal to fibrin while red clots were most common in the LAA subtype compared to the CE source.

**Table 1:** Demographic and clinical characteristics of the patients.

**Factors**	**CE (*n* = 56, 59.6%)**	**LAA (*n* = 38, 40.4%)**	***p***
Overall rate, *n* (%)			
Sex (male), *n* (%)	16 (28.6%)	30 (78.9%)	***P*** **<** **0.001**
Age (y), median (IQR)	76.5 (14)	69 (22)	***P*** **=** **0.009**
Vascular risk factors, *n* (%)			
Hypertension	36 (64.3%)	22 (57.9%)	0.666
Diabetes mellitus	16 (28.6%)	10 (26.3%)	1.000
Hyperlipidemia	2 (3.6%)	4 (10.5%)	0.217
Atrial fibrillation	54 (96.4%)	0 (0.0%)	***P*** **<** **0.001**
Miocardial infarction	15 (26.8%)	7 (18.4%)	0.530
History stroke	14 (25.0%)	5 (13.2%)	0.197
Encephalatrophy	28 (50.0%)	14 (36.8%)	0.291
Smoking	10 (17.9%)	26 (68.4%)	***P*** **<** **0.001**
**Occlusion location**			0.549
MCA	26 (46.4%)	14 (36.8%)	
ICA	17 (30.4%)	13 (34.2%)	
CCA	4 (7.1%)	2 (5.3%)	
BA	5 (8.9%)	3 (7.9%)	
VA	0 (0.0%)	2 (5.3%)	
≥Two vessels	4 (7.1%)	4 (10.5%)	
NIHSS, median (IQR)	15.5 (7)	19 (9)	0.057
**Intervention type**			0.083
Aspiration	4 (7.4%)	6 (15.8%)	
Stent retrieval	16 (29.6%)	4 (11.1%)	
Aspiration +stent retrieval	36 (64.3%)	28 (73.7%)	
**CT**			
Volume (mm^3^), median (IQR)	0.1 (0.10)	0.20 (0.38)	0.092
HU value, median (IQR)	52.0 (10)	48 (9)	**0.010**
**IHC staining cells (**‰**)**			
CD3, median (IQR)	0.45 (1.09)	0.97 (3.25)	0.254
CD20, median (IQR)	0.49 (0.76)	0.43 (0.78)	0.975
CD105, median (IQR)	9.46 (10.44)	16.54 (17.57)	***P*** **=** **0.001**
Actin, median (IQR)	8.16 (12.26)	17.46(7.99)	***P*** **<** **0.001**
**Histopathology n (%)**			**0.009**
Red clots	2 (3.6%)	10 (26.3%)	
Mixed clots	40 (71.4%)	22 (57.9%)	
White clots	6 (10.7%)	4 (10.5)	
Organized clots	8 (14.3%)	2 (5.3%)	

*LAA, large artery atherosclerosis; IQR, interquartile range; CE, cardioembolic; MCA, middle cerebral artery; ICA, internal carotid artery; CCA, common carotid artery; BA, basilar artery; VA, vertebral artery; NIHSS, NIH stroke scale; IHC, immunohistochemistry. Bold text indicates a statistically significant difference with a p < 0.05*.

As shown in Table 2, since the homogeneity of variances assumption is not met, Welch's test was adopted to compare the difference between IHC staining cells and histopathology results. CD 3 levels are significantly different in various thrombus types. After Dunnett's T3 test, CD3 of white clots was significantly lower than mixed clots and organized clots (*p* < 0.05). Actin and CD105 were not significantly different in thrombus histopathology results.

**Table 2:** Comparison of immunohistochemistry (IHC) staining cells in different clot constitutions.

	**Red clots (*n* = 12)**	**Mixed clots (*n* = 62)**	**White clots (*n* = 10)**	**Organized clots (*n* = 10)**	**F**	***P*-value**
Actin	14.66 ± 8.37	11.90 ± 7.43	11.67 ± 7.57	8.52 ± 15.85	20.28	0.636
CD3	1.27 ± 1.53	3.22 ± 7.20^b^	0.27 ± 0.33^a^^c^	8.81 ± 7.52^b^	17.06	0.004
CD20	0.39 ± 0.34	1.38 ± 2.76	14.25 ± 29.41^c^	0.11 ± 0.16^b^	9.06	0.181
CD105	12.89 ± 9.12	11.43 ± 10.29	13.69 ± 5.18	14.15 ± 13.85	24.13	0.709

*Means compared with red clots, p < 0.05*;

a *means compared with mixed clots, p < 0.05*;

b *means compared with white clots, p < 0.05*;

c *means compared with organized clots, p < 0.05*.

**Figure 1:**
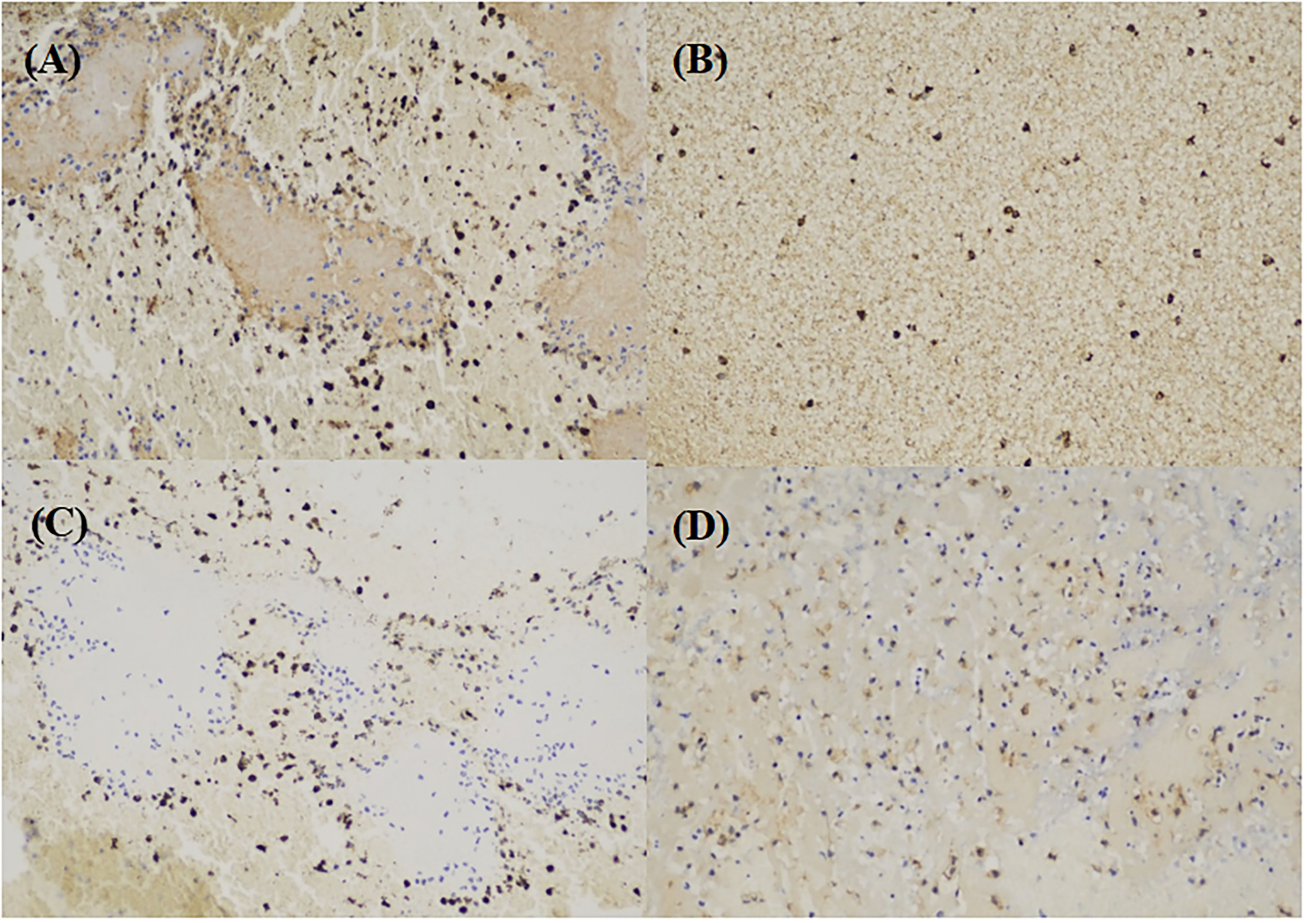
Immunohistochemistry (IHC) imaging. **(A)** actin (brown) in large-artery atherosclerosis (LAA) stroke subtype. **(B)** CD3 (brown) in LAA stroke subtype. **(C)** CD20 cells (brown) in LAA stroke subtype. **(D)** CD105 cells (brown) in LAA stroke subtype.

Immunohistochemistry (IHC) staining imaging is shown in Figure 1. Actin, CD3, and CD105 were randomly distributed in the thrombus, not restricted in the marginal part.

**Figure 2:**
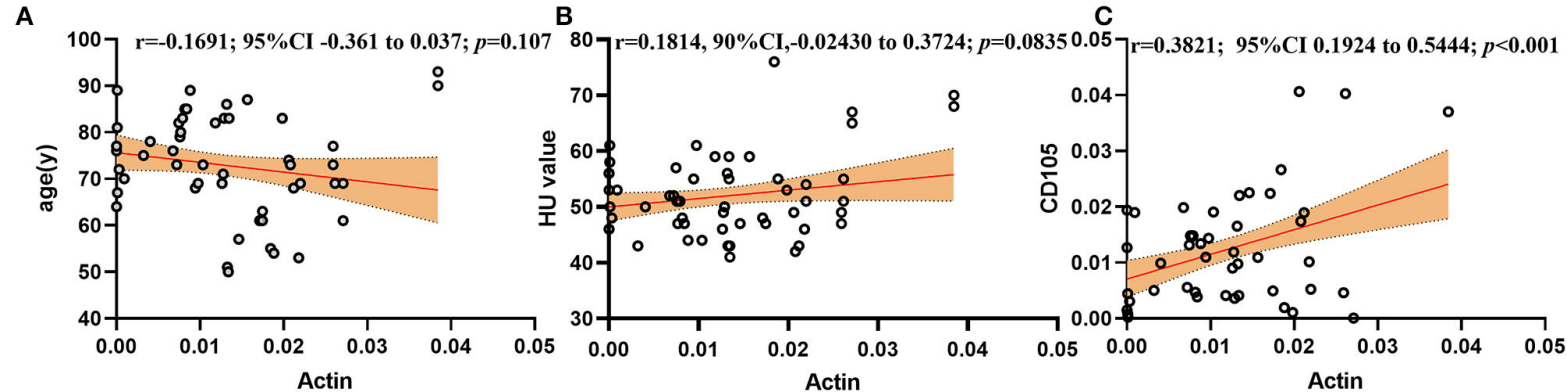
Correlations of the actin concentration with **(A)** age (y), **(B)** HU value, and **(C)** CD105.

In correlation analyses, actin was significantly and positively associated with CD105 (Figure 2C), even after being adjusted for age, sex, intervention type, CD3, CD20, and thrombus density (partial r = 0.379, *p* < 0.001). However, Actin and thrombus density did not show a significant correlation (Figure 2B), even after being adjusted for age, sex, intervention type, CD3, CD20, and CD105 (partial r = 0.184, *p* = 0.090). Actin and age did not show a significant correlation (Figure 2A).

Multivariate analysis shows that actin levels significantly predict the LAA stroke subtype (OR, 1.148; 95% CI, 1.075–1.227; *p* < 0.001), even after being adjusted for age, sex, and intervention type (OR, 1.129; 95% CI, 1.048–1.216; *p* = 0.001), thrombus density and CD105 expression (OR, 1.161; 95% CI, 1.056–1.277; *p* = 0.002; for details, see Table 3).

**Table 3:** Logistic regression analysis for the association of actin with atherosclerotic stroke.

**Variables**	**OR**	**95%CI**	**P**
Unadjust actin	1.148	1.075–1.227	**<0.001**
Model 1	1.129	1.048–1.216	**0.001**
Model 2	1.161	1.056–1.277	**0.002**

*CI, confidence Interval*.

*Model 1 adjusted for age, sex, and intervention type*.

*Model 2 adjusted for Model 1, thrombus density, and CD105 levels*.

*Bold text indicates a statistically significant difference with a p < 0.05*.

**Figure 3:**
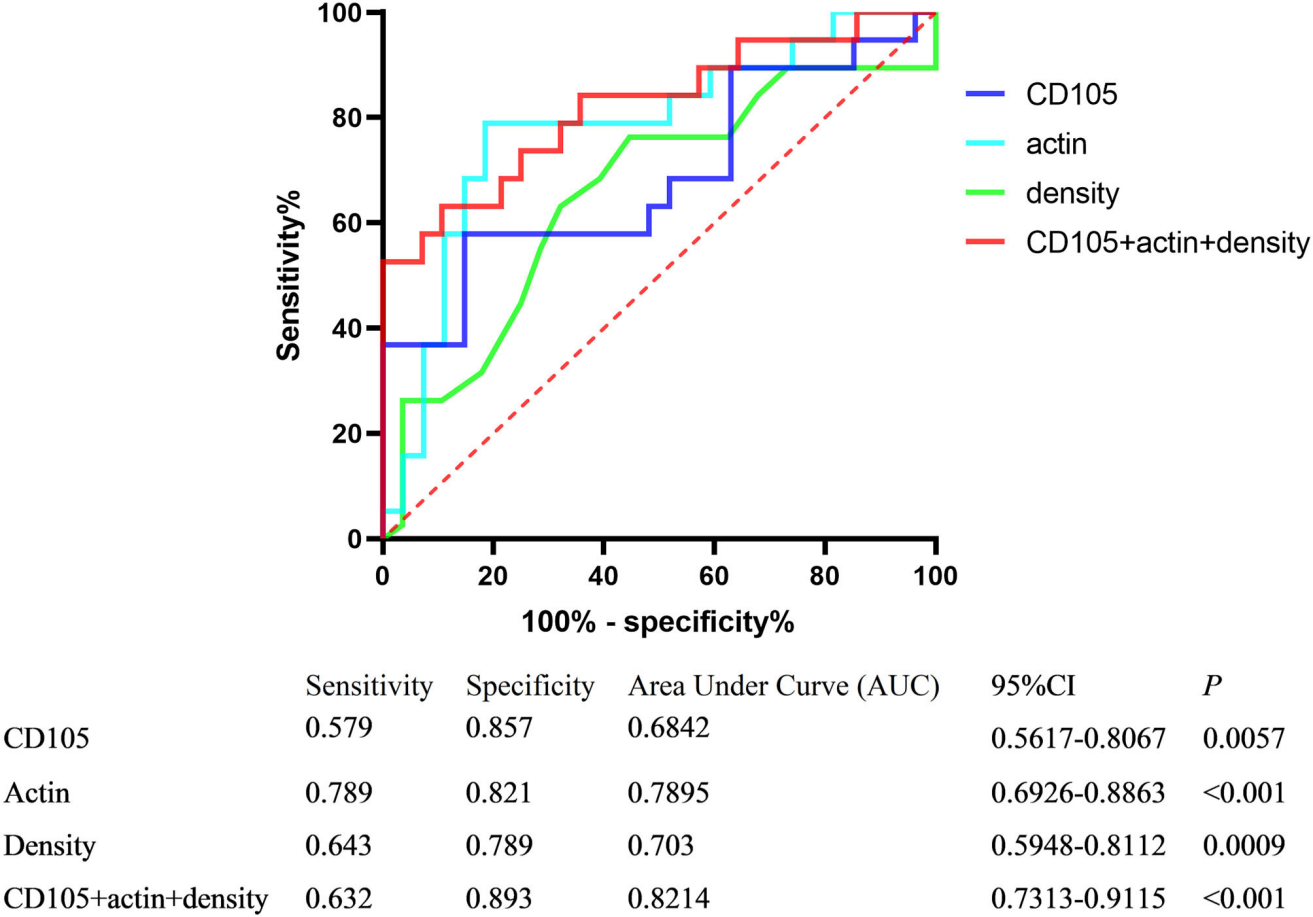
Receiver operator characteristic (ROC) curves of variables differentiating stroke subtypes.

The ROC curve analysis was performed to evaluate the sensitivity and specificity in differentiating stroke subtypes with HU values, CD105, and actin expression of IHC analysis. The areas under the ROC curve were 0.703, 0.684, and 0.789, respectively (Figure 3). A combination of thrombus CT density, CD105, and actin reached the highest frequency of area under the curve (AUC), which yielded a sensitivity of 63.2%, a specificity of 89.3%, a positive predictive value of 85.5%, and a negative predictive value of 70.8%, with the AUC at 0.821 (95% CI, 0.731–0.912).

## Discussion

The clot composition plays a key role in determining sensitivity to thrombus mechanical retrieval and pharmacological lysis as well as the recanalization success rate. The analysis of thrombus composition is vital for the novel targeted lysis therapy and for the improvement of the new devices for thrombus retrieval by its *in vitro* model. As the first choice for suspected patients with AIS, NCCT is also emerging as an essential part of the etiology differentiation and personalized treatment of stroke. Therefore, we performed the correlative study of the NCCT and histopathologic analysis of retrieved thrombus with stroke etiology in AIS to find whether there is a relationship between each other, and whether CT information is associated with both stroke type and thrombus components. It could help in the therapy decision and serve as a useful additional tool to identify patients with occult AF, other cardioembolic risk factors, or hematological disorders for stroke. Unfortunately, we did not find a significant correlation between CT information with stroke etiology and thrombus components. However, we found that the actin levels have a strong ability to differentiate atherothrombotic origin strokes from the cardioembolic stroke.

This study showed that subjects that have an LAA source tend to exhibit higher actin and CD105 levels and lower CT density than subjects with CE. The actin was positively correlated with CD105 and not significantly correlated with thrombus density even adjusted for other confounders. Actin levels were strongly associated with the LAA subtype. A combination of CT density, actin, and CD105 levels has a strong accuracy in discriminating LAA from the CE subtype. This is the first study investigating the actin and CD105 levels in intracranial thrombus retrieved from patients with AIS.

The IHC staining (CD3, CD20, CD105, actin) was performed to explore whether lymphocyte and endothelial cells could give additional information on the origin of the thrombus. However, there were no significant associations except the actin and CD 105 expression between subjects with LAA or CE subtypes in the present study. Even a previous study shows that CD3 was higher in atherothrombotic origin stroke compared with cardioembolic (15). Different histological staining and assessment methods might partly contribute to the different findings. Dargazanli et al. analyzed the CD3 expression by manual counting with the ImageJ software. Additionally, the thrombus retrieved from that study was considerably small.

The IHC staining cells did not significantly differ in thrombus histopathology results except that CD3 is more common in organized clots and CD20 in white clots. Actin levels did not get influenced by different histopathology characteristics.

Previous studies suggest that the level of CD105 and actin is closely related to plaque formation and stability (28, 29). CD105, also known as endoglin, is a membrane-bound glycoprotein that can exist independently on the cell membrane surface. The transforming growth factor-β (TGF-β) receptor complex is its main component, and antagonizing TGF-β inhibits vascular endothelial cells and promotes angiogenesis. As reported by previous studies, high expression of CD105 has been significantly associated with inflammation and angiogenesis, which may result in plaque instability (30–32).

As a cytoskeletal protein that expresses nearly all eukaryotic cells, actin is a major part of the vascular cells' mechanical, organizational, and signaling transformation. The change of actin microfilaments has shown a strong relationship with the reduction of stress fibers and dysfunctional adhesion, causing the accumulation of immune cells and the formation of atherosclerotic plaque (33, 34). Recently studies have also suggested that actin is a vital autoantigen target that mediates the autoimmune process of plaque formation and stability in patients with carotid atherosclerosis (35, 36).

Additionally, substantial evidence indicates that the actin cytoskeleton and its related proteins are susceptible to reactive oxygen species (ROS) (37, 38). Studies in atherosclerosis also showed that ROS modulate cytoskeletal changes in endothelial cells (26, 39). Actin has been proven as a principal part of the endothelial cells' barrier function, interacting with cell adhesive structures, which influence the cells' contraction, shrinking, and formation of blood vessels (40, 41). In this study, the actin and CD105 are randomly distributed in the thrombus, not restricted to one region, which means they are possibly involved in the beginning formation of the thrombus. Moreover, the CD105 is positively associated with the actin levels, which may reflect that endothelial cells may play an essential role in thrombus formation. Vascular dysfunctions caused by pathological changes of vessels such as atherosclerosis, restenosis, and thrombosis could partly lead to vascular diseases, stroke for example (42). Further ROC-curve analysis shows the high accuracy of a combination of thrombus density, CD105, and actin in differentiating the LAA subtype in patients with AIS.

The present study shows that patients with the CE subtype have a higher absolute thrombus attenuation, which is similar to the previous study (16) but in contrast to the study performed by Boodt et al. (43). Differences in results could have been caused by the fact that the latter study included patients suffering from AIS with proximal intracranial vessel occlusion in the anterior circulation. Therefore, their results may not be fully in line with our study of patients with intracranial artery occlusion either in the anterior or posterior. Furthermore, the method differs in the thrombus attenuation measure which may cause this difference. Finally, the reason may lie in the location of the thrombus. MCA occlusion accounting for 46.4% is larger than 36.8% of the LAA group in the CE group. Previous studies have suggested that MCA occlusion thrombus density is significantly higher than those of vertebrobasilar circulation stroke (44).

As reported by the previous studies (5, 7, 8, 45, 46), the present study found that RBC dominant thrombus was significantly associated with atherothrombotic origin stroke. At the same time, mixed clots were more common in patients with the CE subtype, contrary to the study performed by Kim et al. (47). They reported a significantly higher proportion of fibrin and platelets in thrombus originating from large-artery atherosclerosis. Differences in methods and study populations may be the primary reason for this difference. The number of patients with LAA (*n* = 8) was too small to draw a firm conclusion compared with the subjects with CE. In addition, the intravenous recombinant tissue plasminogen activator (rt-PA) given might influence the thrombus composition in their study.

There are several limitations in this study that must be paid attention to. First, for enough patients that could have been be recruited, we did not set rigid restrictions for the occlusion location and Endovascular Treatment (EVT) treatment type. The process of MT and aspiration might influence the thrombus composition to some extent, therefore, the histologic analysis results could have been affected. Second, some of thrombus were fragmented or macerated among all retrieved thrombus due to the retrieval procedure characteristic. Furthermore, the manual segmentation of the thrombus components may be associated with an operator bias.

## Conclusion

Our findings suggest that the level of actin was a major factor differentiating atherothrombotic origin strokes from the cardioembolic stroke.

## Data Availability Statement

The original contributions presented in the study are included in the article/supplementary material, further inquiries can be directed to the corresponding author.

## Ethics Statement

The studies involving human participants were reviewed and approved by Research Ethics Committee of Ya'an People's Hospital. The patients/participants provided their written informed consent to participate in this study.

## Author Contributions

RW contributed to the study design and original draft writing. ZW contributed to the methodology and data curation. LJ contributed to the imaging analysis. GG and BZ performed the histologic analysis. LX and YZ performed the data collection and statistical analysis. JW performed the supervision. All authors reviewed the draft and contributed to the article and approved the submitted version.

## Funding

The authors acknowledge funding from the Sichuan Province Medical Research Project (Nos. S17003) and the Sichuan Science and Technology Department Project (Nos. 2019ZYZF0063; 2 and 2020YJ0497).

## Conflict of Interest

The authors declare that the research was conducted in the absence of any commercial or financial relationships that could be construed as a potential conflict of interest.

## Publisher's Note

All claims expressed in this article are solely those of the authors and do not necessarily represent those of their affiliated organizations, or those of the publisher, the editors and the reviewers. Any product that may be evaluated in this article, or claim that may be made by its manufacturer, is not guaranteed or endorsed by the publisher.

## Abbreviations

AIS: acute ischemic stroke
CE: cardioembolic
LAA: large artery atherosclerosis
HU: Hounsfield Unit
MT: Mechanical thrombectomy
MCA: middle cerebral artery
ICA: internal carotid artery
CCA: common carotid artery
BA: basilar artery
VA: vertebral artery.
